# Physical Exercise and Fitness Level Are Related to Cognitive and Psychosocial Functioning in Adolescents

**DOI:** 10.3389/fpsyg.2020.01777

**Published:** 2020-07-24

**Authors:** Rafael Enrique Reigal, Antonio Hernández-Mendo, Rocío Juárez-Ruiz de Mier, Verónica Morales-Sánchez

**Affiliations:** ^1^University of Malaga, Málaga, Spain; ^2^Department of Social Psychology, Social Anthropology Social Work and Social Services, University of Malaga, Málaga, Spain; ^3^Department of Evolutionary Psychology and Education, University of Malaga, Málaga, Spain

**Keywords:** physical exercise, cognitive, psychosocial, quality of life, health

## Abstract

The purpose of this study was to analyze the relationships among physical exercise and fitness with selective attention, concentration, processing speed, general self-efficacy, self-rated health, and satisfaction with life. 208 adolescents between 14 and 16 years, from the city of Malaga (Spain), participated in the study. A comparative and predictive design was used to carry out this research. The instruments used for the evaluation were the Tanita^®^ BC-601 Body Composition Monitor, the Eurofit Physical Condition Test Battery, the D2 Test of Attention, the WISC-IV Symbol Search and Coding tests, the General Self-Efficacy Scale (GSE), the General Health Questionnaire (GHQ-28), and the Satisfaction with Life Scale (SWLS). Analysis of variance (ANOVA), Kruskal–Wallis test, correlation analysis and linear regression were used to contrast the research objectives. The results indicated that adolescents who practiced more hours of physical exercise per week and were in better physical fitness achieved higher scores in selective attention, concentration, processing speed, general self-efficacy, self-rated health, and satisfaction with life. In addition, cardiorespiratory fitness was the physical fitness variable most closely related to and predictive of cognitive and psychosocial functioning. Cardiorespiratory fitness was predictor of all the variables analyzed, except the factor anxiety and insomnia (self-rated health), and life satisfaction that were predicted by horizontal jump measurements and fat mass, respectively. Thus, the study findings indicate that adolescents who practiced more weekly physical exercise and had a higher level of physical fitness scored better on the cognitive functioning and psychosocial tests evaluated. The data suggest that engaging in physical exercise and fitness in adolescence may be appropriate to improve health and well-being, contributing to better development at this stage.

## Introduction

Numerous studies have pointed out the physical, psychological, and social benefits that physical exercise can bring to adolescents (Esteban-Cornejo et al., 2015; Swann et al., 2018). Among them, the relationships between the practice of physical exercise with cognitive, and psychosocial functioning at these ages are relevant (Eime et al., 2013; Lubans et al., 2016; Cooper et al., 2018; Pontifex et al., 2019). On the one hand, adolescents with adequate cognitive functioning improve their adaptation to the environment, increase the likelihood of success in numerous everyday tasks, and contribute to their future mental health (Lubans et al., 2016; Zmyj et al., 2017). Furthermore, cognitive functioning is related to social cognition, which is essential for effective social interactions (Gil et al., 2012). On the other hand, in adolescence identity is formed, interpersonal bonds are established, and psychosocial skills are developed (Viholainen et al., 2014; Wilson et al., 2014). Physical exercise or sport often takes place in contexts of continuous social interaction and comparison, requiring the acquisition of skills necessary for good psychosocial development (Holt, 2008; Swann et al., 2018). Therefore, practicing physical exercise regularly and in appropriate environments could facilitate better adaptation to the environment, contributing to greater well-being and quality of life (Lubans et al., 2016).

In this context of study, it has been highlighted in recent years that only practicing physical exercise does not ensure health benefits. Some research has pointed to the need to achieve an adequate level of physical fitness to develop improvements in cognitive aspects and optimize psychosocial functioning in people (Herting et al., 2014; Ho et al., 2015; Kantomaa et al., 2015; Fraguela-Vale et al., 2016; Reloba-Martínez et al., 2017). So, various studies have suggested in recent years that cardiorespiratory fitness is the best predictor of optimal cognitive and psychosocial functioning (Becerra-Fernández et al., 2013; Chaddock et al., 2014; Herting et al., 2014; Reigal et al., 2014).

Specifically, in recent years much progress has been made in understanding how physical exercise is related to cognitive functioning. Advances in neuroscientific knowledge (Chaddock et al., 2014), making it possible to resolve questions that were unknown decades ago, are supporting the findings in this area. The use of techniques such as electroencephalography, functional magnetic resonance imaging, and magnetoencephalography, together with the study of proteins such as BDNF (brain-derived neurotrophic factor), IGF-1 (insulin-like growth factor-1), and VEGF (vascular endothelial growth factor), are helping to achieve essential milestones for the development of this field of knowledge (e.g., Tari et al., 2019; Voss et al., 2019). For example, there is evidence of structural changes in the brain associated with increases in physical fitness, and this is a reliable indicator of the impact that physical exercise is thought to have on the brain (Esteban-Cornejo et al., 2017; Chaddock et al., 2018). Also, Chaddock et al. (2010) observed positive relationships among aerobic performance with hippocampal volume and the striated dorsal body. Furthermore, Chaddock et al. (2018) found an increase in the white matter microstructure of the genu of the corpus callosum after a program of physical exercise. In another study, Esteban-Cornejo et al. (2017) noted relationship among cardiorespiratory capacity and speed/agility with the volume of gray matter in various areas of the brain.

Thus, some research has highlighted the relationships among physical exercise and variables such as attention, concentration, working memory, inhibitory control, cognitive flexibility, processing speed, and language (e.g., Liu et al., 2018; Ludyga et al., 2018; Westfall et al., 2018; Xue et al., 2019). Specifically, attention has been an object of interest in a number of studies (Budde et al., 2008; Vanhelst et al., 2016). The ways in which selective attention and concentration are related to physical exercise and physical condition have been analyzed and positive associations have been found between them (Guiney and Machado, 2013; Tine, 2014; Reloba-Martínez et al., 2017). Likewise, cognitive processing speed has been explored, also indicating positive links with physical performance (Hillman et al., 2005; Pontifex et al., 2011). Both selective attention, which is the ability to attend to a particular series of stimuli and ignore others (Giuliano et al., 2014), and concentration or processing speed are factors that affect the efficiency with which multiple tasks are performed, such as academic or social tasks (Perlman et al., 2014; Rabiner et al., 2016).

Also, other studies have pointed out relationships between physical exercise and psychosocial variables such as life satisfaction, self-efficacy, and perception of health. Self-efficacy constitutes the judgments made about one’s own abilities and their effectiveness in carrying out a task (Bandura, 1986). Its development is complex and is thought to depend on factors such as previous successes, vicarious experience, verbal persuasion, and physiological states (Bandura, 1986, 1997). Many authors suggest that self-efficacy is specific; others, however, argue for the existence of general self-efficacy (Schwarzer, 1992; Schwarzer and Jerusalem, 1995). Perception of health refers to the judgments people make about the level of physical or mental health they possess, and it can be a predictor of levels of mortality and future disease, even in a young population (Bombak, 2013; Kantomaa et al., 2015). Finally, satisfaction with life involves an overall assessment of what life itself is like, the conditions in which it is developed, whether the expectations created have been fulfilled or are being achieved, etc., and it is one of the indicators that are usually evaluated when analyzing subjective well-being (Diener et al., 1985; Miller et al., 2019).

The analysis of well-being has been carried out from different models (Ryff, 1989; Keyes et al., 2002). The hedonic tradition presents well-being in subjective terms and is linked to aspects such as life satisfaction. From the eudaimonic tradition it is linked to human potential and has been studied under the construct called psychological well-being (Ryff, 1989). Specifically, Keyes’s model extends Ryff’s to speak of social well-being, and considers that mental health requires positive psychosocial functioning (Keyes et al., 2002). From this point of view, emphasis has been placed on the so-called psychosocial well-being, which would refer to aspects such as perceptions of themselves, the ability to function effectively in the environment or the perception of health (Pinquart and Silbereisen, 2004). For this reason, the analysis of variables such as self-efficacy, perception of health or life satisfaction are relevant to determine the mental health or well-being in people. Therefore, analyze whether physical exercise could be related to these variables becomes very important, due to the repercussion that it can have on the health and quality of life of adolescents.

So, previous research has revealed positive relationships among physical exercise and physical fitness with self-efficacy (Ho et al., 2015), perception of health (Bombak, 2013; Kantomaa et al., 2015), and life satisfaction (Zullig and White, 2011; Fraguela-Vale et al., 2016). In general, appropriate engagement in physical exercise and sport is considered an effective tool for improving numerous psychological issues in adolescence and other stages of life (Lubans et al., 2016).

The aim of this study was to determine if the weekly physical exercise volumen and physical fitness are related to cognitive and psychpsocial functioning in a sample of adolescents. For this purpose, we first analyzed the differences between various groups divided by hours of weekly physical exercise, and secondly, we evaluated whether there were correlations between the study variables, as well as whether physical fitness could predict measures of cognitive functioning and psychosocial variables studied.

## Materials and Methods

### Study Design

This is a comparative and predictive study (Ato et al., 2013).

### Participants

A total of 208 adolescents (boys = 50.96%, girls = 49.04%) aged between 14 and 16 years from the city of Malaga (Spain) were included in the study [mean (*M*) ± standard deviation (*SD*): age = 15.25 ± 0.74 years; height = 167.65 ± 9.67 cm; weight = 63.75 ± 14.76 kg; and body mass index (BMI) = 22.59 ± 4.29 kg/m^2^]. The initial inclusion criteria was to be between 14 and 16 years old. Those who had significant health problems that did not allow them to carry out the evaluation tests following the specified protocol (e.g., physical injury), failed to provide informed consent, or failed to complete the tests correctly were excluded. Out of 224 possible participants, the sample was finally made up of the 208 indicated. Sampling was not probabilistic, it was chosen for convenience.

### Instruments and Measures

(a) D2 Test of Attention (Brickenkamp, 2002). This was used to analyze selective attention and concentration. Performing this test requires discriminating among 47 elements in each of the 14 rows that make up the test (658 elements in total). Each row is completed in 20 s, working from left to right and from top to bottom. The stimuli contain the letters *d* or *p* and may be accompanied by one or two dashes located at the top, bottom, or both. The *d*’s, which are considered relevant stimuli, must be crossed out when they have 2 dashes in any position. The following scores can be obtained: total effectiveness in the test/selective attention index (TOT) and concentration index (CON).
(b) Wechsler Intelligence Scale Coding and Symbol Search tests for children (WISC-IV; Wechsler, 2005). These tests basically assess cognitive processing speed, but also attention, or cognitive flexibility. The Coding test consists of copying a set of symbols, associated with a number, in a certain order. The Symbol Search test involves observing two groups of symbols and indicating whether any of them coincide. The tests have a completion time of 120 s. A Processing Speed Index is obtained from the results.
(c) General Self-Efficacy Scale (Schwarzer and Jerusalem, 1995; Baessler and Schwarzer, 1996; Sanjuán et al., 2000). This consists of 10 items and analyzes the perception of one’s competence to handle a wide range of situations. It is evaluated with scores between 1 (*strongly disagree*) and 10 (*strongly agree*). The internal consistency (Cronbach’s Alpha) value for this study was 0.84.
(d) General Health Questionnaire in its 28-item version (GHQ-28; Goldberg, 1978; Lobo et al., 1986). This was initially designed to assess psychiatric disorders in a community setting and in non-psychiatric clinical settings, although it has subsequently been used for other populations. It is composed of 28 items and explores the following 4 dimensions: somatic symptoms, anxiety and insomnia, social dysfunction, and severe depression. It is answered with scores from 0 (*absence of health problems*) to 3 (*presence of health problems*). The internal consistency (Cronbach’s Alpha) values for this study were somatic symptoms = 0.83, anxiety and insomnia = 0.74, social dysfunction = 0.78, and severe depression = 0.81.
(e) Satisfaction with Life Scale (SWLS; Diener et al., 1985; Atienza et al., 2000). This analyzes life satisfaction and is made up of 5 items. It is answered with scores between 1 (*strongly disagree*) and 7 (*strongly agree*). The internal consistency (Cronbach’s Alpha) value for this study was 0.81.
(f) Anthropometric and physical fitness measurements. To describe the sample, height and weight were analyzed using a conventional measuring rod and scale, respectively. The fat mass percentage was measured with a bioimpedance meter (Tanita^®^ BC-601 Body Composition Monitor). Explosive power in the lower body was evaluated with the horizontal jump test (Eurofit, 1993). Speed was asssessed using the 5 × 10 meter test (Eurofit, 1993). Maximum oxygen consumption was obtained indirectly with the *Course Navette* test (Léger et al., 1988; Eurofit, 1993), an incremental round trip test over 20 meters, increasing the speed by 0.5 km/h every minute from 8.5 km/h. Oxygen consumption was calculated using the formula VO_2_max = 31.025 + 3.238S–3.248A + 0.1536SA, where S is the speed reached in the last completed stage and A is the age of the participant.

### Procedure

The sample was obtained in schools fron the city of Malaga (Spain) by contacting each school and requesting permission for adolescents to participate. In addition, informed consent was obtained from the parents or legal guardians of the participants. Throughout the process, the ethical principles set forth in the Declaration of Helsinki (World Medical Association, 2013) were respected. In addition, this study is part of a line of research that has been positively evaluated by the Ethics Committee of the University of Malaga (No. 243, CEUMA Registry No.: 18-2015-H).

Anthropometric and physical condition evaluations were carried out in Physical Education classes. Cognitive assessment was conducted in a noise-free classroom and in groups. The questionnaires were self-administered and completed in a group, in a normal classroom. In addition, written data were obtained on the number of hours spent each day in physical exercise, using a simple form. Specifically, information was collected on structured physical activity, but not that which was not (e.g., walking to school, shopping, climbing the stairs, etc.). That is, whether it was federated or not, only the information related to the training of a sport or that activity that was carried out with a specific purpose was collected (e.g., running, playing basketball with friends, etc.). On the basis of this information, the sample was divided into three groups: Group 1, low level of physical exercise (less than 2 h per week); Group 2, moderate level of physical exercise (2 to 4 h per week); and Group 3, high level of physical exercise (more than 4 h per week).

### Data Analysis

Descriptive and inferential analyses were performed. The Kolmogorov–Smirnov test was used to analyze the normality of the data. Analysis of variance (ANOVA) were used to assess differences between the groups. Bonferroni statistic would be used to analyze multiple *post hoc* comparisons if there were significant differences between groups. Cohen’s *d* was used to estimate the size of the effect between groups. Correlations were assessed with the Pearson and Spearman coefficients. In order to ascertain the predictive capacity of physical condition for the other variables, linear regression analyses (successive steps) were used (Ruiz-Barquín, 2008). The SPSS computer program, version 20.0, was used for statistical processing.

## Results

### Descriptive Analysis and Normality of Data

Tables 1, 2 show the descriptive statistics and the Kolmogorov–Smirnov test for the total sample and each group, divided by hours of activity per week. The results indicated normality problems in some variables (bold text, Table 2). The ln(x), x2, and 1/x algorithms were used to correct this. All variables were adjusted.

**TABLE 1:** Mean and standard deviation of physical fitness assessment tests and cognitive and well-being indicators.

	Total		Group 1		Group 2		Group 3	
				
	(*n* = 208)		(*n* = 76)		(*n* = 68)		(*n* = 64)	
	*M*	*SD*	*M*	*SD*	*M*	*SD*	*M*	*SD*
FM%	21.57	9.54	27.67	9.14	21.09	7.38	15.04	7.21
HJT	164.86	39.92	144.61	34.35	162.64	35.26	190.41	36.38
VO_2_max	42.93	8.19	37.87	7.01	43.54	6.75	48.15	7.28
5 × 10	18.85	2.24	19.90	2.19	18.84	2.13	17.65	1.80
D2-TOT	59.79	18.73	54.28	17.90	60.27	17.89	65.65	18.89
D2-CON	58.57	20.59	53.00	19.33	59.38	19.68	64.17	21.51
SYM	11.38	2.46	10.78	2.73	11.29	2.15	12.15	2.23
COD	10.09	3.08	9.33	2.99	10.24	2.76	10.80	3.33
PS	105.20	12.21	101.54	13.37	105.53	9.77	109.09	11.91
GSE	6.88	1.97	6.14	2.20	6.88	1.40	7.71	1.87
GHQ-SS	0.74	0.44	0.81	0.49	0.84	0.40	0.57	0.36
GHQ-AI	0.92	0.73	1.04	0.74	1.06	0.71	0.63	0.64
GHQ-SDy	0.93	0.46	0.97	0.53	0.97	0.45	0.83	0.37
GHQ-SDe	0.43	0.53	0.46	0.56	0.51	0.55	0.31	0.47
LS	4.88	1.26	4.51	1.27	4.77	1.09	5.43	1.25

**Abbreviations*: *M*, Mean; *SD*, Standard deviation; FM%, Body fat mass percentage; HJT, Horizontal jump test (cm); VO_2_max, Maximum rate of oxygen consumption (mL/kg/min); 5 × 10, Speed test (s); D2, D2 test; TOT, Total effectiveness in the test/Selective attention index; CON, Concentration index; SYM, Symbol Search; COD, Coding test; PS, Processing speed; GSE, General self-efficacy; GHQ, Health perception questionnaire; SS, Somatic symptoms; AI, Anxiety and insomnia; SDy, Social dysfunction; SDe, Severe depression; and LS, Life satisfaction.*

**TABLE 2:** Skewness, kurtosis, and Kolmogorov–Smirnov statistic of physical fitness, cognitive, and psychological assessment tests.

	Total (*n* = 208)			Group 1 (*n* = 76)			Group 2 (*n* = 68)			Group 3 (*n* = 64)		
				
	*S*	*K*	*K–S*	*S*	*K*	*K–S*	*S*	*K*	*K–S*	*S*	*K*	*K–S*
FM%	0.15	–1.19	1.28	–0.69	–0.39	1.16	–0.01	–1.02	0.96	1.01	–0.32	1.06
HJT	0.25	–0.93	1.57*	1.07	0.72	0.97	0.09	–0.78	0.76	–0.41	–0.46	0.80
VO_2_max	0.14	–1.21	1.54*	1.22	0.06	1.29	0.19	–1.57	1.01	–0.94	–0.36	1.34
5 × 10	0.43	–0.84	1.35	–0.18	–0.57	0.54	0.39	–0.85	0.94	1.54	2.16	1.32
D2-TOT	–0.13	–1.15	1.51*	0.51	–0.92	1.59*	–0.14	–0.94	1.04	–0.90	–0.19	0.98
D2-CON	–0.10	–1.15	1.27	0.38	–0.93	1.29	–0.01	–0.88	1.17	–0.75	–0.70	1.28
SYM	0.29	0.38	1.66**	0.18	–0.44	0.98	0.45	–0.03	0.97	0.92	1.71	1.75**
COD	0.43	0.35	1.48*	0.11	0.00	1.22	0.43	0.92	1.52*	0.67	–0.20	1.19
PS	0.18	0.17	1.51*	0.19	–0.12	0.72	0.19	0.64	0.87	0.52	–0.37	1.18
GSE	–0.33	–0.27	1.16	–0.27	–0.94	1.42*	0.15	–0.12	1.01	–0.29	–0.63	1.13
GHQ-SS	0.43	0.01	1.28	0.38	0.09	0.84	0.26	–0.35	1.04	0.40	–0.74	1.19
GHQ-AI	0.65	–0.26	1.62*	0.38	–0.53	0.83	0.62	0.16	0.71	1.19	0.61	1.53*
GHQ-SDy	0.88	1.61	1.28	1.04	0.84	1.51*	0.81	2.59	0.98	0.00	–0.31	1.03
GHQ-SDe	1.56	2.30	1.82**	1.52	2.35	1.76**	1.52	2.31	1.91**	1.76	2.43	1.49*
LS	–0.14	–1.13	1.59*	0.04	–1.45	1.32	–0.10	–1.07	1.46*	–0.55	–0.63	1.26

*Abbreviations*: *S*, Skewness; *K*, Kurtosis; *K–S*, Kolmogorov–Smirnov statistic; FM%, Body fat mass percentage; HJT, Horizontal jump test; VO_2_max, Maximum oxygen consumption; 5 × 10, Speed test; D2, D2 Test; TOT, Total effectiveness in the test/Selective attention index; CON, Concentration index; SYM, Symbol Search; COD, Coding test; PS, Processing speed; GSE, General self-efficacy; GHQ, Health perception questionnaire; SS, Somatic symptoms; AI, Anxiety and insomnia; SDy, Social dysfunction; SDe, Severe depression; and LS, Life satisfaction. *p < 0.05 and **p < 0.01.

### Inter-Group Mean Differences

The ANOVAs performed indicated that there were differences between the groups in the variables fat mass percentage, horizontal jump test, VO_2_max, 5 × 10 speed test, D2-TOT, D2-CON, Symbol Search, Coding, Processing Speed, general self-efficacy, somatic symptoms, anxiety and insomnia, and life satisfaction. There were no differences between groups in social dysfunction, and severe depression (GHQ). Table 3 shows the comparisons between groups (with Bonferroni correction) for each variable. Furthermore, Levene’s test indicated that there was homegeneity between group variances in each case (*p* < 0.05).

**TABLE 3:** Inter-group (*post-hoc*) comparisons for each variable with significant differences.

	ANOVA				Group 1 vs Group 2 *Sig.*	Group 2 vs Group 3 *Sig.*	Group 1 vs Group 3 *Sig.*
	
	*F*_[2,205]_	*Sig.*	*η2*	1–β			
FM%	43.94	*<0*.001	0.30	0.99	<0.001^1^	<0.001^1^	<0.001^3^
HJT	29.94	*<*0.001	0.23	0.99	<0.01^1^	<0.001^1^	<0.001^2^
VO_2_max	38.26	*<*0.001	0.27	0.99	<0.001^1^	<0.001^1^	<0.001^2^
5 × 10	21.24	*<*0.001	0.17	0.99	<0.01^1^	<0.001^1^	<0.001^2^
D2-TOT	6.92	*<*0.01	0.06	0.92	—	—	<0.001^1^
D2-CON	5.49	*<*0.01	0.05	0.85	—	—	<0.01^1^
SYM	5.86	*<*0.01	0.05	0.87	—	—	<0.01^1^
COD	4.29	*<*0.05	0.04	0.74	—	—	<0.05^1^
PS	7.20	*<*0.01	0.07	0.93	—	—	<0.001^1^
GSE	11.49	*<*0.001	0.10	0.99	—	<0.05^1^	<0.001^1^
GHQ-SS	8.06	*<*0.001	0.07	0.96	—	<0.01^1^	<0.01^1^
GHQ-AI	8.18	*<*0.001	0.07	0.96	—	<0.01^1^	<0.01^1^
LS	10.67	*<*0.001	0.09	0.99	—	<0.01^1^	<0.001^1^

**Abbreviations.* FM%, Body fat mass percentage; HJT, Horizontal jump test; VO_2_max, Maximum oxygen consumption; 5 × 10, Speed test; D2, D2 Test; TOT, Total effectiveness in the test/Selective attention index; CON, Concentration index; SYM, Symbol Search; COD, Coding test; PS, Processing speed; GSE, General self-efficacy; GHQ, Health perception questionnaire; SS, Somatic symptoms; AI, Anxiety and insomnia; and LS, Life satisfaction. Cohen’s *d*: ^1^*d* > 0.20; ^2^*d* > 0.40; and ^3^*d* > 0.60.*

### Correlation and Linear Regression Analysis

Tables 4, 5 shows the correlations between measures of physical fitness and cognitive functioning. There were significant relationships among them. Maximum oxygen consumption was the measure of physical fitness that best correlated with the measures of cognitive functioning.

**TABLE 4:** Correlation between measures of physical fitness and cognitive functioning.

	D2		WISC-IV		
		
	TOT	CON	SYM	COD	PS
FM%	−0.20**	−0.19**	−0.32**	−0.21**	−0.31**
HJT	0.23**	0.22**	0.31**	0.19**	0.29**
VO_2_max	0.36**	0.32**	0.38**	0.21**	0.35**
5 × 10	−0.17*	−0.16*	−0.26**	−0.16*	−0.24**

*Abbreviations.* FM%, Body fat mass percentage; HJT, Horizontal jump test; VO_2_max, Maximum oxygen consumption; 5 × 10, Speed test; D2, D2 test; TOT, Total effectiveness in the test/Selective attention index; CON, Concentration index; SYM, Symbol Search; COD, Coding test; and PS, Processing speed. *p < 0.05 and **p < 0.01.

**TABLE 5:** Correlation between measures of physical fitness and psychosocial variables.

	GSE	GHQ				LS
		
		SS	AI	SDy	SDe	
FM%	−0.34**	0.32**	0.29**	0.17*	0.11	−0.32**
HJ	0.33**	−0.37**	−0.35**	−0.17*	–0.12	0.30**
VO_2_max	0.41**	−0.46**	−0.34**	−0.14*	−0.19*	0.31**
5 × 10	−0.23**	0.32**	0.32**	0.15*	0.08	−0.24**

*Abbreviations.* FM%, Body fat mass percentage; HJT, Horizontal jump test; VO_2_max, Maximum oxygen consumption; 5 × 10, Speed test; GSE, General self-efficacy; GHQ, Health perception questionnaire; SS, Somatic symptoms; AI, Anxiety and insomnia; SDy, Social dysfunction; SDe, Severe depression; and LS, Life satisfaction. *p < 0.05 and **p < 0.01.

Table 6 shows the linear regression analyses (successive steps) with which we attempted to identify the physical fitness variables that predict the values of the psychological measures analyzed. The models meet the assumptions of linearity in the relationship between predictor variables and criteria, homoscedasticity, and normal distribution of residuals whose mean value is 0 with a standard deviation of almost 1 (0.99). The Durbin–Watson value was between 1.60 and 2.05, which is appropriate according to Pardo and Ruiz (2005), indicating that it can be assumed that the residuals are independent and the assumption of independence of the independent variables with respect to the dependent variable is met. The models obtained included a single variable, in most cases maximum oxygen consumption (Table 6).

**TABLE 6:** Linear regression analysis (successive steps).

*R*	*R*^2^ corrected	D-W	Criterion variable	Predictor variable	Standardized beta	T	VIF
0.34	0.11	1.62	D2-TOT	VO_2_max	0.34	1.00	1.00
0.30	0.09	1.60	D2-CON	VO_2_max	0.30	1.00	1.00
0.39	0.15	1.89	SYM	VO_2_max	0.39	1.00	1.00
0.21	0.04	1.96	COD	VO_2_max	0.21	1.00	1.00
0.35	12	1.95	PS	VO_2_max	0.35	1.00	1.00
0.37	0.14	1.72	GSE	VO_2_max	0.37	1.00	1.00
0.50	0.24	1.96	GHQ-SS	VO_2_max	−0.50	1.00	1.00
0.35	0.12	2.05	GHQ-AI	HJT	−0.35	1.00	1.00
0.19	0.03	1.72	GHQ-SDy	VO_2_max	−0.19	1.00	1.00
0.18	0.03	1.86	GHQ-SDe	VO_2_max	−0.18	1.00	1.00
0.32	0.10	1.62	LS	FM%	−0.32	1.00	1.00

**Abbreviations.* D2, D2 test; TOT, Total effectiveness in the test/Selective attention index; CON, Concentration index; SYM, Symbol Search; COD, Coding test; PS, Processing speed; GSE, General self-efficacy; GHQ, Health perception questionnaire; SS, Somatic symptoms; AI, Anxiety and insomnia; SDy, Social dysfunction; SDe, Severe depression; LS, Life satisfaction; VO_2_max, Maximum oxygen consumption (mL/kg/min); HJT, Horizontal jump test (cm); FM%, Body fat mass percentage; T, Tolerance; and VIF, Variance Inflation Factor.*

## Discussion

The purpose of this study was to analyze how physical exercise and physical fitness are related to certain cognitive and psychosocial functioning variables. The results obtained show relationships between the measures studied and therefore meet the objective of the research. Specifically, the data reveal differences in the measures evaluated in favor of adolescents who performed more hours of physical exercise per week, as well as significant associations of physical fitness with cognitive functioning and the various psychosocial indicators.

Firstly, we observed that adolescents who were physically active for a larger number of hours achieved higher scores in measures of cognitive functioning, specifically selective attention, concentration, and speed of processing. This is in line with previous studies that identify a relationship between physical exercise and these measures in the adolescent population (Hillman et al., 2005; Pontifex et al., 2011; Guiney and Machado, 2013; Tine, 2014; Reloba-Martínez et al., 2017). In addition, it is noteworthy that there were no differences between adolescents with low and moderate levels of physical exercise (Groups 1 and 2). That is, only those who engaged in a high number of hours of activity per week (Group 3) showed significant differences from those with lower levels. This coincides with previous research that draws attention to the need for physical exercise to attain a certain degree of intensity and frequency in order to produce functional brain changes (Herting et al., 2014; Reloba-Martínez et al., 2017).

There is therefore no guarantee that doing a certain number of hours of physical exercise per week or at a certain intensity will explain this phenomenon, so to understand it better we need to monitor the level of physical fitness achieved. In this study, the groups into which the sample was divided had different levels of physical fitness, and the group that engaged in the most hours of physical exercise per week achieved the best results. This is consistent with the arguments of authors such as Chaddock et al. (2018) or Esteban-Cornejo et al. (2017), who consider that improvement in physical fitness is an suitable indicator to explain cognitive changes. It is also in line with other data obtained, which show that measures of physical fitness are significantly correlated with attention, concentration, and processing speed. Specifically, cardiorespiratory fitness, assessed by indirect calculation of maximum oxygen consumption, was a particularly significant value; indeed, it was the main predictor in the linear regression models. This connects with other studies which identity this variable as the one that best explains cognitive functioning (Chaddock et al., 2014; Herting et al., 2014; Reloba-Martínez et al., 2017).

As described in previous research, physical exercise contributes to brain plasticity. In other words, it helps the brain to be more prepared to change its functioning. Possibly only exercise does not produce the change, but it does produce greater sensitivity for it to occur. And for this, a series of physiological phenomena occur so that the brain is more willing to be modified. However, for this to occur a high impact on the body must be produced, which would be reflected in the level of fitness reached by the person (Chaddock et al., 2010, 2018; Esteban-Cornejo et al., 2017). That is why, in recent years, the data supports that physical exercise of moderate and high intensity and frequency would better explain the changes produced in the functioning of the brain. In addition, these studies show that exercises that increase cardiorespiratory fitness, cause greater synthesis of biomolecules such as BDNF or IGF-1, and facilitate volume increase in cortical and subcortical gray matter (Esteban-Cornejo et al., 2017; Tari et al., 2019).

Secondly, differences between the groups in our study according to the amount of physical exercise undertaken indicate that the most active and fittest group achieved the best scores for psychosocial indicators. These results support previous findings that highlight this phenomenon in populations of similar ages (Eime et al., 2013; Lubans et al., 2016), and specifically they are consistent with other work that assesses general self-efficacy, perception of health, and satisfaction with life (Zullig and White, 2011; Bombak, 2013; Ho et al., 2015; Kantomaa et al., 2015; Fraguela-Vale et al., 2016).

Furthermore, this previous research indicates that physical fitness was a determining factor in assessing the effects of physical exercise on these psychological variables. It is pertinent to point out that in our study some dimensions of the GHQ, such as social dysfunction or serious depression, showed no differences between the groups divided according to hours of physical exercise per week. However, when correlation and linear regression analyses were performed, physical fitness was shown to be significantly related to these factors. This coincides with the arguments put forward in other studies, which consider it necessary to assess physical fitness to better understand how physical exercise relates to these types of variables (Reigal et al., 2014). In addition, cardiorespiratory fitness also emerges, in most cases, as the physical fitness factor most closely related to the other variables, which is in line with what has been described in other studies (Becerra-Fernández et al., 2013).

Probably, the relationship between physical exercise, fitness and psychosocial functioning is justified for multiple reasons. Among them, when physical exercise is performed, personal skills are increased. Not only the physical ones, which is obvious, but also those derived from the social context in which it develops. Thus, when doing physical exercise, you have to interact with other people and you have to learn to improve social skills. Therefore, learning would be generated that would affect the perception of personal competence. In addition, there is an improvement in physical health, but also a greater subjective feeling of well-being caused by enjoyment with the activity that is performed.

This work has some limitations. First, the sample was made up of both boys and girls, but the analyses have not been differentiated by gender for each group. Future research should seek to verify whether the results are similar for each gender. Second, the design employed does not allow for the establishment of causal relationships. Thus, quasi-experimental or longitudinal designs could provide valuable information about changes in the variables studied due to the practice of physical exercise. Finally, it would be interesting in subsequent research to assess the relationships between measures of cognitive and psychosocial functioning, given that more complete profiles could be established on how these variables are associated with each other. In any case, the results of this study provide valuable information on the relationships between physical exercise, physical fitness, and cognitive and psychosocial functioning in adolescents, which suggests the need to continue promoting the practice of physical exercise among the young population.

## Conclusion

The study findings indicate that adolescents who practiced more weekly physical exercise and had a higher level of physical fitness scored better on the cognitive functioning and psychosocial tests evaluated. Besides, cardiorespiratory fitness was predictor of all the variables analyzed, except the factor anxiety and insomnia (self-rated health), and life satisfaction that were predicted by horizontal jump measurements and fat mass, respectively. Among others, if increasing the level of physical exercise and fitness can affect their cognitive and psychosocial functioning, it would be contributing to having a healthier life and increasing their ability to adapt to the daily demands of life. Thus, adolescents must face multiple situations, such as academic ones, social interactions, etc., that could be favored by the continued practice of physical exercise. For all this, and referring to the evidence collected on this subject, it is necessary to indicate that despite the large amount of leisure time activities availables to adolescents today, active lifestyle habits are likely to be among those that bring the most direct and indirect benefits to their health and well-being. Therefore, any effort to develop this type of behavior will have been worthwhile.

## Data Availability Statement

The raw data supporting the conclusions of this article will be made available by the authors, without undue reservation.

## Ethics Statement

The studies involving human participants were reviewed and approved by University of Malaga. Written informed consent to participate in this study was provided by the participants’ legal guardian/next of kin.

## Author Contributions

AH-M, VM-S, RJ-R, and RR participated in the study design and data collection, performed the statistical analyses, contributed to the interpretation of the results, wrote the manuscript, approved the final manuscript as presented, reviewed and provided feedback to the manuscript, and made substantial contributions to the final manuscript. All authors contributed to the article and approved the submitted version.

## Conflict of Interest

The authors declare that the research was conducted in the absence of any commercial or financial relationships that could be construed as a potential conflict of interest.
